# Justice and Equity Implications of Climate Change Adaptation: A Theoretical Evaluation Framework

**DOI:** 10.3390/healthcare4030065

**Published:** 2016-09-07

**Authors:** Melanie Boeckmann, Hajo Zeeb

**Affiliations:** 1 Sustainability Research Center (artec), University of Bremen, Enrique-Schmidt-Str. 7, Bremen 28359, Germany; 2 Department Prevention and Evaluation, Leibniz Institute for Prevention Research and Epidemiology—BIPS, Achterstr. 39, Bremen 28359, Germany; zeeb@leibniz-bips.de; 3 Health Sciences Bremen, University of Bremen, Bremen 28359, Germany

**Keywords:** climate change, Public Health, social inequalities, environmental health, ethics, adaptation, environmental justice

## Abstract

Climate change affects human health, and climate change adaptation aims to reduce these risks through infrastructural, behavioral, and technological measures. However, attributing direct human health effects to climate change adaptation is difficult, causing an ethical dilemma between the need for evidence of strategies and their precautionary implementation before such evidence has been generated. In the absence of conclusive evidence for individual adaptation strategies, alternative approaches to the measurement of adaptation effectiveness need to be developed. This article proposes a theoretical framework and a set of guiding questions to assess effects of adaptation strategies on seven domains of health determinants, including social, economic, infrastructure, institutional, community, environmental, and cultural determinants of health. Its focus on advancing gender equity and environmental justice concurrently with the implementation of health-related adaptation could serve as a template for policymakers and researchers.

## 1. Introduction: Climate Change Affects Human Health

Climate change is expected to adversely affect human health through direct and indirect paths. Direct effects of global environmental change include increased exposure to extreme weather and temperature events, comprising heat waves, cold spells, storms, and floods [1,2,3]. Indirect effects are mediated through natural and social systems [1]. Natural systems mediate changes in disease vector distribution, increased air pollution, and pollen distribution, as well as a higher frequency of food- and water-borne infections. Social systems, on the other hand, influence indirect climate effects on crop production and distribution, mental health, and occupational health and safety [1]. Worldwide and within societies, exposure risks and vulnerability to negative health effects of climate change are unequally distributed [1,4,5,6]. Beyond the natural distribution patterns of climate-related hazards, humans’ abilities to withstand shocks and the extent of damages caused by these hazards are determined by social, cultural, and economic capital and power [7,8,9,10,11,12], making climate change and its related policies—beyond other concerns—an ethical issue [4].

Using extreme heat events as an example, studies have shown that risks of heat stress, heat stroke, or excess cardiovascular and respiratory mortality during extreme events are often increased for people living in inner city, heat island-prone areas [13], for the elderly [14], and for persons suffering from pre-existing conditions [15]. Different susceptibilities linked to gender [16] and ethnicity [17] have also been suggested. The distribution of risk in higher income populations resembles that of broader health and illness risk patterns in conditions affected by social determinants, linking climate change vulnerabilities to health vulnerabilities via their susceptibility to social disadvantages [18,19]. Within the climate change and health discourse, the concept of climate justice [6,20,21] mirrors environmental justice’s conviction that environmental hazards and exposures disproportionately affect people with lower socio-economic capital, people of color, people in lower income regions or city districts, children, and people with less political power [22].

Climate change adaptation strategies aim at reducing risks to human health from effects of inevitable changes [2], and adaptation policies target not only environmental, but also public health and healthcare sectors. Typical examples of adaptation measures include flood protection, awareness and information campaigns, heat warning systems, and disease surveillance and monitoring [23]. The effects of individual measures have not yet been conclusively proven [24,25], however, and potentially adverse outcomes generated by adaptation are still under-researched [26].

Considering the above mentioned unequally distributed vulnerabilities to adverse effects, evaluation approaches that specifically incorporate or address these aspects might be useful [27,28].

## 2. The Need for an Alternative Framework: Evaluation Problems and the Precautionary Principle

Proving effects of specific adaptation measures on health outcomes has been challenging within epidemiology. As with many complex public health interventions, the medical hierarchy of evidence, ranking from meta-analyses and randomized controlled trials (RCTs) to case reports, cannot quite account for the multiple paths and factors that interact with a structural intervention project such as adaptation [29], challenging our understanding of their causality.

### Causality as an Evaluation Problem

In his theory on risk and causality, Alex Broadbent [30,31] puts forward the following points: (a) the conceptual work on causal inference is not finished; (b) the difference between cause and association needs to be examined in more detail; (c) the restrictions of epidemiological study design make finding a clear link highly difficult; and (d) these issues are not sufficiently discussed in epidemiological discourses nor in philosophy of science, even though the topic has gained wider attention among epidemiologists and other scientists in the last decade [32]. Extending this conceptual work on causal inference to climatic factors and health risks, it follows that the epidemiological understanding of causality reaches its limit when applied to climate change adaptation and health outcomes. The mechanisms through which climate change indirectly affects health are hypothesized but not conclusively “proven” in the strict definition of the word. Consequently, neither have the mechanisms through which adaptation to climate change indirectly affects health. Previously acknowledged problems in climate change and health effect evaluation research include how to deal with unmeasured confounders and control groups when the exposure is climate-related, a lack of pre-tests where heat warning plans were already established before research took place, and a lack of studies examining interactions between social, clinical, and environmental factors [25,33]. Using natural experiments to evaluate adaptation as a complex intervention could be one solution [34], and is delivering first promising results [35]. However, as Craig et al. [34] describe, natural experiments in policy areas on “wider determinants of health” might lead to ambiguous results, and the Grading of Recommendations, Assessment, Development and Evaluations (GRADE) ratings in systematic reviews of complex interventions have been shown to inadequately describe their evidence base [24,36]. Beyond quantitative assessments, qualitative and process evaluations have gained increased traction in adaptation research [37,38]. Public Health research on adaptation continues to struggle with epistemological differences between epidemiological effectiveness definitions and the application of qualitative research paradigms [39], in which studies often do not aim at reaching statistically generalizable conclusions but rather to explore phenomena in depths [40].

Without conclusive evidence for effectiveness of adaptation, policymakers are faced with two main options: to (a) proceed with adaptation implementation under the precautionary principle [41,42]; and (b) re-assess the notion of effectiveness in light of the health protection goals that adaptation is supposed to reach.

The precautionary principle has previously been connected to climate change and health research [43], and is well-suited to enable decision-making in situations of high uncertainty and high risks. The precautionary principle serves as justification for implementing adaptation before evaluation results have been shared [44]. As a next step, the evaluation challenges need to be addressed. Based on the challenges of associating climate change adaptation to health outcomes, the proposed theoretical framework uses proxies of adaptation effects on social and cultural determinants of health.

## 3. Results: A Theoretical Framework for Adaptation Evaluation Based on Social and Environmental Health Determinants

The underlying principle of the proposed framework is an adaptation strategy’s potential for climate justice that serves as proxy indicator for effectiveness in adaptation evaluation. Our definition of climate justice refers to distributional justice [27] of the burden of adverse health effects, closely linked to the concept of environmental justice in the Public Health context. Environmental justice is based on the notion that environmental hazards and exposures disproportionately affect people with lower socio-economic capital, people of color, people in lower income regions or city districts, children, and people with less political power [22]. Within the climate change and health discourse, the concept has been extended to climate justice [6,20,21]. The latter similarly argues that the greatest risks of climate change consequences are shouldered by those who have contributed the least to these increased risks [45]. While mostly discussed at an international level and focused on responsibilities of higher income nations with large greenhouse gas emission profiles towards lower income nations [20,45], climate justice is also a local issue [6]. With regard to public health, the possible adverse health effects caused by climatic change are also expected to be unequally distributed [1].

Adaptation to climate change has the potential to protect human health [46], but the decision to implement adaptation requires that a need for protection be recognized. As such, climate change adaptation is highly reliant on normative assessments of responsibility for health protection. These links between responsibility, climate justice, and adaptation evaluation for human health can be addressed by focusing on a normative understanding of public health’s role in advancing social justice [47], and could contribute to new evaluation approaches in health and climate change adaptation research.

**Figure 1:**
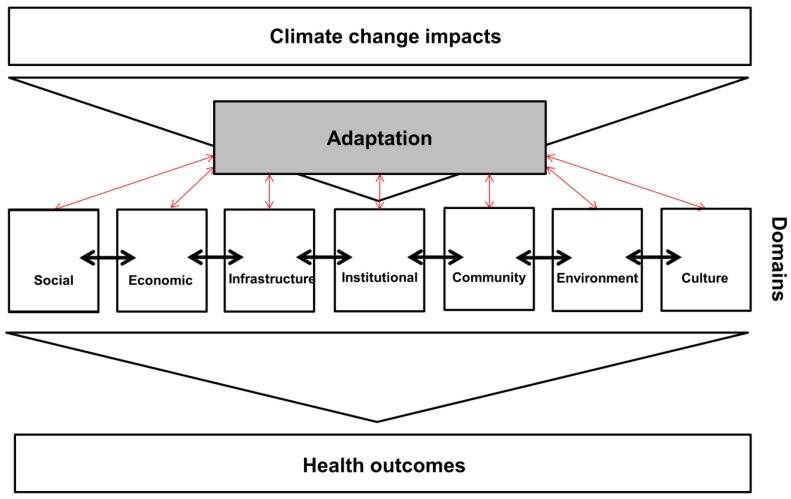
The domain-driven theoretical framework to evaluate adaptation based on justice concerns. Evaluation projects should target the associations between adaptation and each of the seven domains.

The aim of this proposed framework is to aid in assessing whether adaptation targets current inequities and determinants of health that may reduce unequally distributed health outcomes. To evaluate health-related climate change adaptation for its climate justice contribution, the theoretical framework draws from two main concepts: (i) health inequity [48,49,50] and (ii) resilience to climatic changes [51,52]. In more detail, these two concepts can be illustrated using seven domains (Figure 1), which illustrate variables and contents with which to measure possible climate justice advancement. Susan Cutter et al. [53] describe six of these domains which are relevant to disaster resilience: social, economic, infrastructure, institutional, community, and environmental domains. As current research shows that culture can limit or facilitate “opportunities to become healthier” [54], we propose adding culture as a further domain. Together, these seven domainns cover the range of likely determinants of human health potentially affected by climate change. They serve as the overarching evaluation frame. Table 1 supplements this framework by providing an overview over possible operational indicators for each area (adapted from [6,53,54]).

Climate change affects each of these spheres, represented by the top large arrow in Figure 1. Additionally, each domain has impacts on the others through multiple pathways. These interactions are important representations of contextual interdependencies of health determinants, for instance between education and health [55], or between social and economic determinants [56]: individual factors within domains as well as combined effects of two or more domains affect health outcomes.

In this framework, adaptation is positioned between climate change and the seven domains, and acts as a mediator of climate change effects on the determinants of health. At the same time, societal developments within each of these fields influence adaptation scope, design, implementation, and effects. The paths between adaptation and the seven domains are therefore the targets of evaluation in this framework: as a proxy for direct effects of climate change adaptation on health outcomes (which are difficult to measure), effects of adaptation on the seven domains can be assessed.

It has to be noted that these domains were created for purposes other than adaptation evaluation. Cutter et al. [53] applied their framework to assessing resilience, and Napier et al. [54] were interested in the role of culture as a health determinant. However, since climate change as a “wicked problem” [57] systemically affects human-made and natural environments, factors that contribute to risks of illness are also of relevance in the climate change context.

Within the social domain, gender-related indicators are of high importance as they are acknowledged as highly relevant [58,59], yet remain under-theorized in climate change adaptation research output [60,61,62]. The roles of intersectionality and gender in climate change assessments are still ill-defined and mostly limited to discussions on vulnerability differences between women and men [61]. This extends to climate change, adaptation, and health research, and is unsurprising considering the debates on integration of intersectionality concepts and gender-sensitive approaches to data collection and analysis in social epidemiology [63,64,65]. Despite these difficulties, increasing awareness of gender as a category worth exploring in adaptation evaluation promises greater applicability of strategies to different population groups [66].

Overall, using this framework helps add the dimension of health inequities to adaptation evaluation approaches. It aims to fill the gap of limited evidence of adaptation effectiveness through its focus on determinants of health rather than on specific health outcomes that are usually at the center of adaptation assessments in public health research [67,68].

## 4. Framework Application

The framework can serve as a template for interdisciplinary research involving environmental, cultural, social, and health researchers. Adaptation planners could also profit from application of this framework to assist them in design and evaluation of measures. Policymakers and researchers can choose indicators from each domain most relevant to their research question. The framework stresses the role of determinants of health in climate adaptation, and targets the connections between adaptation and the domains as opposed to the less easily assessable associations between adaptation and health outcomes.

What could such an evaluation process look like? Table 2 presents guiding questions to assess potential impacts of measures taken. In brief, each domain and indicator need to be conceptualized for the specific context and community by (a) assessing the status quo, and (b) contemplating what changes to which the adaptation measure might lead. The framework requires careful conceptualization of the questions to ask to be able to provide a guide for adaptation design and evaluation. The guided questions listed here serve merely as a starting point that should be amended and added to as befits the particular situation. Situation specific response categories can be developed beforehand, such that applicability by different persons, professions and institutions in a country can be secured. Additionally, all adaptation work depends on prior risk and impact analysis within the specific context and situation. Results of these analyses will then help further refine the question template. These questions can only be thoroughly answered by applying a combined approach of strategy document analysis and interviews. As several of these questions require an in-depth analysis of context and target groups, for example, the questions on safety of green spaces or participation processes, they will ideally be refined and answered jointly by stakeholders and researchers.

### Example: Applying the Framework to an Adaptation Action Plan

To illustrate possible use of the framework, we tested selected guiding questions on the Austrian Climate Change Adaptation Action Plan document [69] as the Austrian strategy has been identified as a good contribution to health protection from climate change [46]. Only one question for each domain was asked here to keep the case study brief for information purposes. In a “real world” case study, the example questions listed here would be adapted to specific contexts and evaluation aims. In addition, as stated above, not all questions can be adequately answered using a document analysis approach. Further tests in actual adaptation projects and the use of expert information are needed. Table 3 summarizes our case assessment.

## 5. Discussion

This framework relies on a normative framing of climate change and health in which distributional climate justice and reduced adverse health effects are desired outcomes of adaptation. Harriet Bulkeley et al. [6] propose adding the notion of recognition to the concept of climate justice, which links socio-economic to cultural injustice [6]. This notion in turn applies the concept of climate justice to sub-national and local contexts as well. Likewise, the role of social and cultural determinants on human health has long been recognized in Public Health [49], and needs merely to be extended and reframed for the climate change era. The framework thus combines previous research on environmental justice and health determinants [70,71] and links it with climate change aspects. Where the influence of social, environmental, and cultural determinants on human health is acknowledged, this framework helps link these influences to climate change adaptation. Justice can therefore be promoted through reduced health inequities with society overall, not only if linked directly to climate change adaptation. Climate justice has developed into an important movement that has much to offer to Public Health [72]. Major requests posed by the climate justice community, such as increase participation of diverse population groups [21], gender equality in representation [73], financial support to those suffering most from adverse effects [45], and procedural rights in the political climate change arena [72], are chances to remain healthy as well [74]. Learning from these movements can therefore support adaptation and health research and practice. Our proposed framework wishes to establish synergies between justice efforts and Public Health efforts.

Each of the seven domains and their respective indicators could be analyzed with different foci, thus allowing for conscious inclusion of intersectionality, for instance. As the roles of gender and diversity in climate adaptation have not yet been sufficiently explored, particularly in higher-income contexts [59,62], their contribution to climate justice through adaptation could be considered. Gender equality is a normative goal that has long been important to Public Health [75], and is becoming more prominent in climate change politics as well [76]. Sex and gender relations are also established health determinants [77,78]. Intersectionality as an analytical tool and a perspective can be used to assess “how structures of power emerge and interact” [57] (p 418). A low-threshold approach could be to ask questions to gain insights into additional, alternative knowledge that would otherwise stay hidden, as has been proposed by Kaijser & Kronsell [60] for the field of climate change in general, and illustrated in our framework specifically for health-related adaptation. More concretely, this can entail asking about gender relations when faced with an issue related to race, or about class when faced with an issue about gender relations [60,79]. Using the framework always requires digging deeper into what structures and powers may be affected by the proposed strategies. This focus on political processes [80] is useful when analyzing adaptation as most of these measures stem from top-down approaches and environmental governance. As the proposed framework serves to support the assessment of patterns of health determinants and effects of adaptation on these determinants, the questions that intersectionality frameworks elicit can support recognition of these patterns.

## 6. Challenges to Framework Application

Our proposed approach assumes awareness of likely causal paths between health determinants and health outcomes. Its practical application relies on data availability and collaboration between health and environment sectors, as well as between practitioners and academia. Critical assessment of the concept through testing it in actual adaptation evaluation situations is necessary to refine the guiding questions for each context. Nonetheless, as a first template, the framework serves both policymakers and researchers to link ethics to the climate change and health discourse.

The framework supports awareness of interactions between health determinants. While it points out the importance of assessing effects on gender relations, it does not in itself solve the complexity of deconstructing intersectional layers. The challenges of breaking theoretical concepts of equity and justice down into operationalized indicators are persistent in adaptation research [81]. The framework can, however, support critical thinking on these issues and encourages multi-disciplinary teams to accept and tackle complexity in climate change adaptation.

Reducing inequities and risk factors for health disparities does not eliminate the need to further develop epidemiological methods to measure health outcomes after adaptation. Instead, this framework expands the notion of effectiveness to allow for an assessment at an early stage in adaptation implementation: changes to determinants of health are likely detected earlier than effects on specific health outcomes—particularly since the pathways between climate change adaptation and health effects are multi-faceted and long-term.

## 7. Conclusions

This article showed that the problem of adaptation evaluation is linked to challenges in establishing causality and to inadequate definitions of adaptation’s effectiveness in protecting human health. The theoretical framework puts forward the idea of using a set of proxy indicators, measuring effects of adaptation on determinants of health in seven domains. As such, the framework allows for measures to reduce health-related inequities and to advance climate justice to become part of the adaptation evaluation standard.

To complement this framework, standard epidemiological studies to identity causal relationships are still needed. The key is to view both the methodological and the theoretical approaches as useful. This framework can help to circumnavigate the limitations of causality outlined above, but it cannot replace the standard definition of effectiveness. Instead, combined approaches, such as applying epidemiological methods to analyze indicator relationships within the domains, will likely increase applicability of the model.

The framework is not intended to be static. Researchers should test and further develop the model, and make their findings public to increase awareness about the role of climate justice in health and adaptation research.

Can climate justice become a Public Health ethics issue for the anthropogenic climate change era? While the two have been linked, the role of justice in adaptation and specifically health-focused adaptation has rarely been discussed. This comes as a surprise, particularly in light of the long research tradition in Public Health focusing on the social, environmental, and cultural determinants of health. Climate change is fraught with ethical implications, such as vulnerability to increased weather extremes, fuel poverty, access to clean air, and safe housing. This theoretical framework acknowledges these connections and aims to provide a template to be filled with contextually appropriate variables in evaluation.

## Figures and Tables

**Table 1:** Possible operational indicators for each domain (adapted from [6,53,54]).

Domain	Selected Indicators
Social	Gender relations
	Education
	Ethnicity
Economic	Income
	Employment
Infrastructure	Health services
	Built environment
	Access to information (e.g., Internet access)
Institutional	Local governance
Community	Civic engagement
Environment	Green spaces
	Exposure
Culture	Cultural construction of health
	Values

**Table 2:** Guiding questions to conceptualize the framework indicators (example questions).

Domain	Indicator	Suggested General Questions Related to Indicators
Social	Gender equity	Does the adaptation measure require special attention to gender relations? For example, does the measure require unpaid care work to support older persons or children during extreme temperature events? Is the measure targeting only one gender, and, if so, do data support such an approach? Is the language used, i.e., in information material, gender-sensitive?
	Education	Does the adaptation measure require a specific type of knowledge and experience that the target group might need to acquire? For behavior change adaptation, are instructions suitably formulated and distributed for all targeted groups? Are language and distribution channels used in the measure appropriate for the target groups?
	Ethnicity	Is there diversity within the target group? Are the adaptation materials targeting specific ethnic groups, and, if so, why? Do data support such an approach? Is the adaptation measure, i.e., information material, perpetuating stereotypes? Are materials culturally appropriate? Who is creating and disseminating the adaptation measure: how diverse is the team?
Economic	Income	What are potential effects of the adaptation measure on income equality? Could the proposed adaptation strategy require target groups to spend money, i.e., for electricity or new household items?
	Employment	Will the adaptation measure change provide more income opportunities through new jobs, i.e., in green technology or in adaptation policy? Will socially disadvantaged populations have the opportunity to profit from these new jobs? Are potentially targeted professions, i.e., nurses, included in strategy design?
Infrastructure	Health services	Is the health system sufficiently prepared and staffed to respond to emergencies, i.e., during heat events or floods? Are the distances to health services longer for vulnerable groups than for others? Can the targeted groups get health-related adaptation information easily through widely accessible channels?
	Built environment	Does the built environment pose a risk from climate change impacts, i.e., through dense concrete buildings during heat? Does the built environment pose obstacles to effective adaptation, i.e., fear of crime, inaccessible or expensive to access green or cool spaces?
	Access to information	Can all targeted groups regularly access relevant information about the adaptation measure, i.e., via TV, radio, or the Internet? How frequently is information about measures distributed? Are potential language barriers among target groups taken into account?
Institutional	Local governance	How is the involvement of affected groups into local decision-making organized?
Community	Civic engagement	Are affected communities involved in planning or implementing of the adaptation measure?
Environment	Green spaces	Are green spaces safely accessible to all targeted groups?
	Exposure	Are some groups disproportionately affected by environmental hazards such as waste, air pollution, or polluted waters? Is there a disaster risk reduction plan that has been well communicated to communities at risk?
Culture	Cultural construction of health	Does the adaptation measure account for different perceptions of health risks and vulnerabilities in the affected communities?
	Values	How does the adaptation measure consider multiple societal values about the environment and human health?

**Table 3:** An example of using suggested questions to assess a strategy document: The Austrian Adaptation Action Plan [69].

Domain	Indicator	Suggested Questions Related to Indicators	Sections of Strategy Document Replying to Questions
Social	Gender equity	Does the adaptation measure require special attention to gender relations? For example, does the measure require unpaid care work to support older persons or children during extreme temperature events?	Volunteer work to support older persons during heat events is recommended: no reflection on who these volunteers might be is available in document (p. 224) [69]. Might indicate effects on gender relations.
	Education	Does the adaptation measure require a specific type of knowledge and experience that the target group might need to acquire?	Information campaign on health effects of extreme events and infections is planned to be tailored to target groups: dissemination via social media for youths, as part of teaching curricula, and via TV and radio for adults (p. 221) [69].
	Ethnicity	Are the adaptation materials targeting specific ethnic groups, and, if so, why?	No specific mention of ethnicity. Might be included in “hard to reach target groups”, further analysis is necessary (p. 220) [69].
Economic	Income	What are potential effects of the adaptation measure on income equality?	No information provided. An analysis of the workforce structure in targeted fields could yield pointers on potential effects.
	Employment	Will the adaptation measure change provide more income opportunities through new jobs, i.e., in green technology or in adaptation policy?	The outlined measures will provide additional opportunities in existing fields, i.e., in infectious disease monitoring or drinking water monitoring. Whether additional resources will be provided is unspecified (p. 228 for water) [69].
Infrastructure	Health services	Is the health system sufficiently prepared and staffed to respond to emergencies, i.e., during heat events or floods?	Additional need to further educate health personnel on climate change risks is acknowledged (p. 221) [69]. No information on health system preparedness is given: might indicate a need for better cross-sectoral integration.
	Built environment	Does the built environment pose a risk from climate change impacts, i.e., through dense concrete buildings during heat?	This risk is acknowledged. It is planned to disseminate information on regional, cool spaces with easy access for use during heat events (p. 221) [69].
	Access to information	How frequently is information about measures distributed?	No information on frequency of general awareness campaign. Heat warnings are disseminated during heat events (p. 220) [69].
Institutional	Local governance	How is the involvement of affected groups into local decision-making organized?	No information provided. Could be included in strategy for sector other than health. Comprehensive analyses of all sectors targeted in a strategy beyond health could yield further information.
Community	Civic engagement	Are affected communities involved in planning or implementing of the adaptation measure?	Calls for volunteers are issued regarding post-disaster reconstruction, for example (p. 228) [69]. The extent of participation is not clear.
Environment	Green spaces	Are green spaces safely accessible to all targeted groups?	No information provided.
	Exposure	Is there a disaster risk reduction plan that has been well communicated to communities at risk?	Flood risk management plans exist (p. 227) [69]. Their dissemination is not specified.
Culture	Cultural construction of health	Does the adaptation measure account for different perceptions of health risks and vulnerabilities in the affected communities?	The document outlines different vulnerabilities (p. 221) but does not link these to cultural differences or perception differences [69].
	Values	How does the adaptation measure consider multiple societal values about the environment and human health?	The document portrays scientific values in repeated calls for data collection (i.e., p. 235 on plant and pollen changes) [69]. Societal diversity is targeted only in relation to vulnerability.
